# The Host Immune Response to Enterovirus A71 (EV-A71): From Viral Immune Evasion to Immunopathology and Prognostic Biomarkers of Severe Disease—A Narrative Review

**DOI:** 10.3390/v17121540

**Published:** 2025-11-25

**Authors:** Anna Andronik, Dawid Lewandowski, Artur Sulik, Kacper Toczylowski

**Affiliations:** Department of Pediatric Infectious Diseases, Medical University of Bialystok, Waszyngtona 17, 15-274 Bialystok, Poland

**Keywords:** enterovirus A71 (EV-A71), hand-foot-and-mouth disease (HFMD), immune evasion, immunopathology, cytokine storm, biomarkers, neurotropism, adaptive immunity

## Abstract

Enterovirus A71 (EV-A71) is a critical global pathogen, primarily causing Hand-Foot-and-Mouth Disease (HFMD) but frequently leading to severe neurological complications, including fatal neurogenic pulmonary edema (PE). This review elucidates the complex interplay between viral pathogenesis and the host immune response. EV-A71 utilizes receptors like SCARB2 and PSGL-1 for entry, while its proteases (2A^pro^, 3C^pro^) efficiently evade innate immunity by cleaving key signaling adaptors (MAVS, TRIF), suppressing Type I IFN response. Critical to disease progression is the age-dependent vulnerability in infants and the subsequent shift toward immunopathology. Severe disease is driven by a systemic cytokine storm and T cell dysregulation, characterized by a loss of control from Treg cells and a profound Th17/Treg imbalance, resulting in high levels of pathogenic cytokines (e.g., IL-17A, IFN-γ). Clinical progression is predicted by specific biomarkers, including Treg depletion, monocyte exhaustion (PD-1/PD-L1), and suppressed regulatory signaling (low cAMP). These findings highlight that effective therapeutic strategies must target host-mediated damage through immunomodulation (e.g., by exploring interventions against key pathogenic axes like IL-6 and IL-1β) and call for the development of next-generation vaccines capable of eliciting balanced cellular immunity to prevent immunopathology.

## 1. Introduction

Enteroviruses are a group of non-enveloped, single-stranded RNA viruses belonging to the Picornaviridae family. They are primarily transmitted through fecal–oral routes, respiratory secretions, or direct contact, often thriving in unsanitary conditions. Enterovirus A71 (EV-A71) is a significant member of this group, first isolated in 1969, and is recognized as a major causative agent of hand, foot, and mouth disease (HFMD), predominantly affecting children under the age of five [1,2].

EV-A71 holds particular clinical importance due to its neurotropic properties and epidemiological impact [3]. While most infections result in mild symptoms such as fever, rash, and oral ulcers, EV-A71 can cause severe neurological complications, including encephalitis, aseptic meningitis, acute flaccid paralysis, and neurogenic pulmonary edema [4]. These complications can be life-threatening, especially in pediatric populations. Epidemiologically, EV-A71 is responsible for large HFMD outbreaks, especially in the Asia-Pacific region, with increasing incidence in Europe attributed to emerging subgenotypes. Severe cases and fatalities, although rare, underscore its public health threat [1,2,5,6].

## 2. Characteristics of Enterovirus A71

To better understand the pathogenic potential of EV-A71 and its neurotropic behavior, it is essential to first outline its structural and molecular characteristics.

### 2.1. Viral Structure and Life Cycle

**Figure 1:**
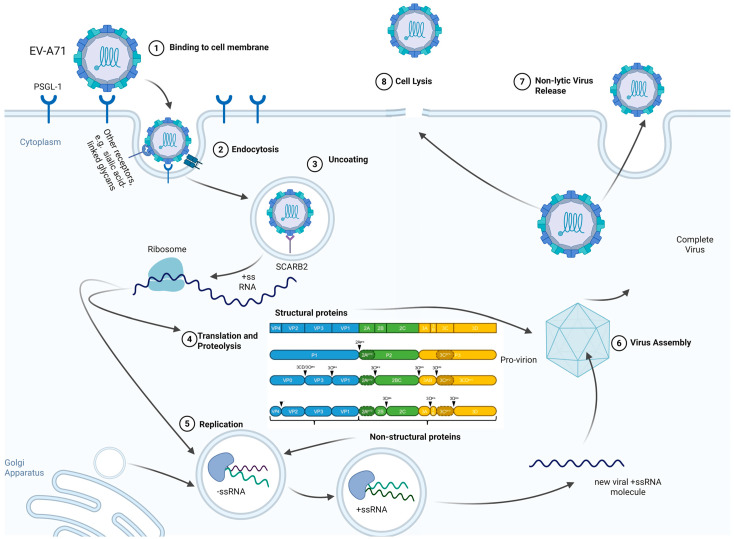
**The Replication Cycle and Key Host Factor Interactions of Enterovirus A71 (EV-A71).** The canonical life cycle of Enterovirus A71 involves several steps orchestrated between the virus and the host cell machinery. Binding and Entry involve attachment to primary host cell receptors, notably SCARB2 and PSGL-1, triggering receptor-dependent endocytosis. The subsequent Uncoating requires the acidic environment of the endosome and is assisted by specific host factors, such as the lipid modifying enzyme PLA2G16, which is crucial for pore formation and the release of the positive-sense RNA genome into the cytoplasm. Following entry, the viral RNA genome, containing an Internal Ribosome Entry Site (IRES), is translated into a single polyprotein. This polyprotein is processed by viral proteases (2A^pro^ and 3C^pro^) into structural and non-structural proteins. 2A^pro^ plays a key role in promoting viral translation by suppressing host cap-dependent synthesis. Viral Replication occurs on virus-induced membranous Replication Organelles (ROs). In motor neurons, these ROs are specifically found to assemble on mitochondrial membranes, suggesting a neuronal-specific mechanism. Replication success also depends on host factors like the methyltransferase SETD3, which interacts with 2A^pro^ and is critical for viral RNA synthesis. Progeny components then assemble into new virions, which are released either through cell lysis or non-lytically via extracellular vesicles.

EV-A71 virions are approximately 24 to 30 nm in diameter and possess an icosahedral capsid composed of 60 copies of four structural proteins: VP1, VP2, VP3, and the internal VP4. These proteins are arranged with a pseudo T = 3 symmetry, a common architecture among enteroviruses [6,7,8]. The viral genome is a single-stranded positive-sense RNA of about 7.4 kilobases, featuring a 5′ untranslated region (UTR) containing an internal ribosome entry site (IRES), a large open reading frame encoding a single polyprotein, and a 3′ UTR with a poly A tail [7,8,9]. The life cycle begins with viral attachment to host cell receptors such as scavenger receptor B2 (SCARB2) and P-selectin glycoprotein ligand-1 (PSGL-1), which mediate viral entry via receptor-dependent endocytosis (Figure 1) [7,8,10,11]. In addition to SCARB2 and PSGL-1, several other host cell receptors have been identified for EV-A71, including sialic acid-linked glycans, annexin A2, heparan sulfate glycosaminoglycans, prohibitin, and tryptophanyl-tRNA synthetase (hWARS) [12,13]. These receptors mediate viral attachment and endocytosis. SCARB2 plays a central role in uncoating the virus in acidic late endosomes. The uncoating process is initiated by the displacement of a stabilizing lipid molecule (“pocket factor”) from a hydrophobic pocket within the capsid upon receptor binding, which triggers a conformational change in the capsid and formation of an expanded intermediate (A-particle) that facilitates genome release [14]. This uncoating process is facilitated by the acidic environment of the endosome and relies on specific host factors, such as the lipid modifying enzyme PLA2G16, which is crucial for pore formation and genome release into the cytoplasm [15]. Following entry into the host cell, the positive-sense RNA is translated into a polyprotein, which is subsequently cleaved by the viral proteases 2A^pro^ and 3C^pro^ into structural and non-structural proteins. Viral replication primarily occurs on rearranged membranous structures; however, in motor neurons, EV-A71 replication organelles (ROs) were specifically found to assemble on mitochondrial membranes, suggesting a neuronal-specific mechanism. Viral replication also depends on host factors such as the methyltransferase SETD3, which interacts with the 2A protease and is critical for viral RNA replication [15]. Assembly of progeny virions involves the formation of pentamers from VP0, VP1, and VP3, which assemble into the capsid encapsulating the genomic RNA, followed by maturation through the cleavage of VP0 into VP2 and VP4. Virions are released either by host cell lysis or non-lytically via extracellular vesicles [16,17].

### 2.2. Tissue Tropism with Emphasis on the Central Nervous System (CNS)

EV-A71 exhibits notable neurotropism, being the most neurotrophic non-polio enterovirus identified to date [18,19]. It can infect neurons and glial cells, as its primary receptors SCARB2 and PSGL-1 are expressed in these cell types [19]. The virus is capable of breaching the blood–brain barrier (BBB) through multiple hypothesized routes, including infection of cerebral microvascular endothelial cells, retrograde axonal transport via peripheral motor nerves, and Trojan horse mechanisms involving peripheral immune cells [20,21,22]. This neuroinvasion often results in rhombencephalitis (brainstem encephalitis) with a stereotyped distribution of inflammation, most intensely affecting the spinal cord anterior horn cells, brainstem, and cerebellar dentate nucleus [18].

Autopsy studies have revealed inflammatory responses in the spinal cord gray matter, brainstem, hypothalamus, subthalamic nucleus, and dentate nucleus [8,20]. Beyond receptor expression, intracellular host factors also contribute to the neurotropism and pathogenesis of EV-A71. One such factor is prohibitin (PHB), a mitochondrial scaffold protein that was recently identified as a key modulator of EV-A71 neurovirulence. PHB is upregulated during infection in motor neuron-like NSC-34 cells, where it facilitates virion binding and entry, as well as the formation of viral replication complexes on mitochondria. Inhibition of PHB using the anti-cancer compound Roc A destabilized mitochondria, reduced ATP production, and significantly impaired viral replication. In vivo, treatment with Roc A delayed the onset of neurological symptoms, prolonged survival, and reduced viral loads in both the spinal cord and brain. These findings suggest that PHB plays a pro-viral role in EV-A71 neuropathogenesis and may represent a novel therapeutic target in severe CNS infection [15,23]. In addition to host–cell determinants of neurotropism, viral genetic variability also strongly influences disease severity and tissue specificity.

### 2.3. Genotype—Pathogenicity Associations in Human EV-A71 Infections

Enterovirus A71 (EV-A71) genotypes differ in their virulence and neurotropism, and specific capsid protein mutations have been linked to clinical disease severity in humans. Among these, the amino acid residue at position 145 of the VP1 protein plays a key role. Most clinical isolates from patients with neurological complications harbor glutamic acid (E) at VP1-145, a substitution that enhances viral interaction with human receptors such as SCARB2 and PSGL-1. In contrast, the Q145 variant, which binds more strongly to heparan sulfate proteoglycans (HSPGs), is more commonly associated with attenuated or animal-adapted strains. Human intestinal and airway organoid models have shown that VP1-145E variants have higher infectivity, suggesting a selective advantage for neuroinvasion in human hosts. Other mutations, such as VP2-I149K, have been associated with reduced infectivity in neural cell lines and may play a role in modulating neurovirulence [24,25,26]. These findings indicate that EV-A71 evolves under selective pressures in human tissues, and that specific capsid mutations, particularly at VP1-145, are important determinants of tissue tropism, immune evasion, and disease outcome in human infection.

## 3. Innate Immune Response to EV-A71

The host innate immune system provides the first line of defense against EV-A71 infection. Its ability to recognize viral RNA and mount interferon responses determines whether the infection remains localized or progresses to systemic and neurological disease.

### 3.1. Early Recognition and Evasion of Host Immune Sensors

The initiation of the host innate immune response to EV-A71 relies on both direct viral entry and intracellular recognition of viral components. Two major mechanisms underlie this early detection: binding to cellular receptors that mediate viral entry, and recognition of viral pathogen-associated molecular patterns (PAMPs) by host pattern recognition receptors (PRRs). These initial interactions influence the virus’s tissue tropism, replication dynamics, and the magnitude of the innate immune response.

Cell surface receptors such as SCARB2 and PSGL-1, described earlier as key mediators of EV-A71 entry, also influence early innate immune responses by determining viral tropism and modulating immune cell infection [15]. SCARB2, a lysosomal membrane protein also expressed on the surface of neural and immune cells (e.g., plasmacytoid dendritic cells), serves as the primary receptor for all EV-A71 strains. It binds to viral capsid proteins VP1 and VP2 via the α5 and α7 loops, triggering uncoating and viral entry via clathrin-mediated endocytosis. In vivo studies using human SCARB2-transgenic mice have demonstrated its essential role in viral neurotropism and the development of neurological symptoms. Additionally, host genetic polymorphisms in SCARB2 have been associated with differential susceptibility and disease severity in HFMD [27].

In contrast, PSGL-1 (P-selectin glycoprotein ligand-1) is a secondary receptor expressed mainly on immune cells, including T cells and pDCs (plasmacytoid dendritic cells). Its interaction with EV-A71 is strain-dependent and facilitates viral entry via caveolae-mediated endocytosis. PSGL-1 may facilitate viral dissemination via infected immune cells and contribute to immune evasion by allowing the virus to infect antigen-presenting cells. Its dual role as a receptor and immunomodulatory molecule places it at the interface of viral pathogenesis and innate immunity. Once inside the host cell, EV-A71 is recognized by intracellular PRRs (pattern recognition receptors) that detect viral RNA. Key intracellular sensors involved include Toll-like receptors (TLRs) and RIG-I-like receptors (RLRs) [15]. TLR3, located in endosomal membranes, detects double-stranded RNA (dsRNA) intermediates formed during replication. Its activation induces type I interferon (IFN) and proinflammatory cytokine production via TRIF-dependent pathways. TLR7, also endosomal, senses single-stranded RNA (ssRNA) and activates the MyD88 pathway, leading to IFN-α production, especially prominent in plasmacytoid dendritic cells (pDCs). MDA5 (melanoma differentiation-associated protein 5) and RIG-I (retinoic acid-inducible gene I) are cytoplasmic sensors that detect viral RNA species with distinct structural features. MDA5 serves as the main cytoplasmic sensor for EV-A71, initiating MAVS-dependent signaling and leading to a strong type I IFN response [28,29]. While both RIG-I and MDA5 may contribute to viral RNA sensing in cells that express both receptors, MDA5 appears to play the dominant role during EV-A71 infection, with RIG-I playing a less prominent, possibly complementary, function [15,28]. Notably, ectopic expression of TLR4 in HEK293 cells enables the detection of EV-A71 virus-like particles and the subsequent induction of the proinflammatory cytokine IL-8, suggesting its potential role in DC activation, despite not being a primary entry receptor [10].

In addition to direct viral sensing via PRRs, host cells may also respond to endogenous danger signals released by infected or dying cells. Damage-associated molecular patterns (DAMPs), such as mitochondrial DNA and HMGB1, can activate pattern recognition receptors such as TLR9 in adjacent, uninfected cells. This has been observed in EV-A71 infection, where TLR9-mediated sensing of DAMPs induces an antiviral immune state in bystander cells, thereby limiting viral spread and protecting tissue integrity [30]. Importantly, EV-A71 has evolved multiple strategies to evade innate immune recognition and suppress type I interferon responses, including protease-mediated cleavage of key adaptor molecules and downregulation of TLR expression [28,31,32]. In response to infection, the EV-A71 2A protease has been shown to reduce the TLR3 protein level in neuroblastoma cells, and is responsible for its subsequent cleavage [10].

### 3.2. Type I and III Interferons and Proinflammatory Cytokines

Enterovirus A71 (EV-A71) initially establishes infection in the intestinal mucosa in most cases, where it targets intestinal epithelial cells (IECs) that form the structural and immunological barrier against enteric pathogens. The mucosal immune system, composed of densely arranged IECs and intraepithelial lymphocytes (iIELs), constitutes the first line of defense, orchestrating local antiviral responses. Upon infection, EV-A71 activates distinct cytokine and interferon pathways within the gut epithelium that are critical for controlling early viral replication and shaping the downstream immune response [33].

Notably, EV-A71 infection in IECs predominantly induces type III interferons (IFNs), specifically IFN-λ1 and IFN-λ2/3, rather than type I IFNs (IFN-α and IFN-β) [33,34]. Studies using human IEC lines (e.g., FHC and HT29) demonstrate that viral infection triggers robust upregulation of IFN-λ transcripts and proteins, while type I IFNs remain minimally expressed [34]. This preferential induction is orchestrated via the TLR3/IRF1 signaling axis: TLR3 detects viral double-stranded RNA, while IRF1 acts as a key transcription factor promoting type III IFN expression. Experimental knockdown of either TLR3 or IRF1 significantly reduces IFN-λ production and enhances viral replication, underscoring their pivotal role in mucosal antiviral defense [34].

Although type I IFNs are less induced in IECs during EV-A71 infection, they retain systemic antiviral capacity. However, the virus has evolved mechanisms to antagonize their function. EV-A71 proteases 2A^pro^ and 3C^pro^ cleave and inactivate key adaptor molecules in the type I IFN induction pathways, including RIG-I, MAVS, and TRIF. This impairs downstream activation of IRF3 and NF-κB, ultimately suppressing type I IFN production and blunting early host responses [35].

Specifically, the 3C^pro^ protease binds and cleaves the TRIF adaptor to inhibit TLR3 signaling towards type I IFN induction [10]. Furthermore, the 2A^pro^ protease directly targets and cleaves the MAVS adaptor at multiple distinct sites, none of which can activate type I IFN production [29,36]. To hinder JAK-STAT signaling, 3C^pro^ EV-A71 cleaves the transcriptional factor IRF9 [29,36]. Additionally, the 2A^pro^ protease may degrade the IFNAR1 receptor, thereby inhibiting STAT1 and STAT2 phosphorylation [29].

Type III IFNs, on the other hand, effectively induce an antiviral state in IECs by stimulating the expression of interferon-stimulated genes (ISGs), such as ISG15, ISG54, PKR, and OAS, which collectively inhibit viral replication. Comparative studies show that recombinant IFN-λ more effectively suppresses EV-A71 replication in IECs than IFN-β. In vivo, systemic administration of IFN-λ2 to infected neonatal mice reduces the viral load across multiple tissues, mitigates tissue damage, and improves survival—highlighting the protective function of type III IFNs in intestinal and systemic compartments [34,35].

In parallel to interferon responses, EV-A71 infection elicits the release of various proinflammatory cytokines and chemokines that modulate disease progression. Elevated levels of IL-6, IL-8, TNF-α, and CXCL10 have been associated with both intestinal and systemic manifestations [37]. IL-6 is elevated in the cerebrospinal fluid (CSF) of patients with enterovirus-induced central nervous system (CNS) inflammation, particularly in brainstem encephalitis (BE) and encephalomyelitis (EM), although its levels appear to be more closely related to patient age than to disease severity [38]. Despite this, animal models show that IL-6 neutralization improves survival, underscoring its pathogenic role. Similarly, increased IFN-γ levels observed in severe cases may contribute to immune-mediated neurotoxicity [39].

The enhanced production of proinflammatory cytokines such as IL-1β may be driven by the activation of the NLRP3 inflammasome in response to EV-A71 infection, with NLRP3 deficiency leading to increased susceptibility in vivo [29].

Temporal profiling of cytokines in patients further distinguishes mild from severe HFMD. In mild cases, early and transient elevations of IL-8, IL-10, and IL-18, alongside a timely IgM response, reflect efficient viral control. Conversely, in severe cases, cytokine peaks are delayed and prolonged, often accompanied by suppressed IL-8 and IL-18 levels, indicative of impaired innate immunity. Elevated IL-10 and IL-13 levels in these patients have been associated with increased vascular permeability and respiratory complications [39,40].

In summary, EV-A71 infection activates a multifaceted immune response in the intestinal mucosa, dominated by type III IFN production through the TLR3/IRF1 axis. This response is critical for viral containment at the entry site. Simultaneously, the virus undermines type I IFN signaling to promote its dissemination. The accompanying release of proinflammatory cytokines and chemokines shapes disease outcomes and may serve as biomarkers for severe forms of EV-A71 infection. Understanding this interplay offers valuable insight into mucosal immunity and potential therapeutic targets for EV-A71-associated disease.

### 3.3. Natural Killer Cell Responses and Monocyte Dysfunction in EV-A71 Infection

Following the initial interferon-mediated antiviral defense, cellular components of innate immunity such as monocytes and natural killer cells play a central role in viral control and immunopathogenesis. The initiation of the host innate immune response against EV-A71 relies on the engagement of phagocytic cells, such as macrophages, and Natural Killer (NK) lymphocytes [41]. In the context of EV-A71 pathogenesis, monocytes/macrophages represent critical early responders, exhibiting a dual role by initiating antiviral defense while simultaneously contributing to virus-induced neural damage [41,42]. EV-A71 infection stimulates macrophage polarization toward the pro-inflammatory M1 phenotype, which is essential for mounting an effective antiviral response but is also directly implicated in neurological pathology. This activation is cascade-like and leads to the further engagement of B cells, NK cells, and T cells [41,42]. Significantly, monocyte status serves as a prognostic factor in HFMD severity. Clinical severe cases show a low absolute count of monocytes, reduced Monocyte Chemoattractant Protein-1 (MCP-1) secretion, and a diminished activation status (HLA-DR, CD38) [43]. It is suggested that in advanced disease, monocytes enter a state of immune exhaustion, indicated by the upregulated expression of the co-inhibitory signals PD-1/PD-L1 [43]. Importantly, in vitro PD-L1 blockade has been shown to partially restore monocyte function and inhibit viral replication [43]. The inflammation phenomenon is further exacerbated by the release of HMGB1 (High-Mobility Group Box 1) from activated macrophages [44]. High extracellular HMGB1 levels trigger a pro-inflammatory cytokine cascade and are pivotal in the pathogenesis of acute lung injury and leading to lethal endotoxemia [44].

In terms of early protection, invariant Natural Killer T (iNKT) cells play a crucial protective role against EV-A71 in young, immunologically immature hosts [45]. iNKT activation is uniquely dependent on TLR3 signaling in macrophages (rather than dendritic cells), requiring both IL-12 production and the presentation of endogenous lipids via the CD1d molecule on these macrophages [45,46]. Deficiencies in either CD1d or TLR3 correlate with more severe disease and increased viral load in the central nervous system [45], underscoring the mechanism by which iNKT cells prevent neuroinvasion.

## 4. Adaptive Immune Response to EV-71

The adaptive immune system is essential for long-term protection against EV-A71, providing specificity and immunological memory [15]. However, strong evidence suggests that both the humoral and cellular arms of immunity can contribute to either protection or pathogenesis in severe HFMD [47]. Clinical studies indicate that cellular immunity, rather than humoral immunity, is a better predictor of disease progression [48].

### 4.1. Humoral Response

The humoral response (NtAb) is crucial for viral neutralization and is the main focus of current licensed vaccines. Neutralizing antibodies (NtAb) appear rapidly after infection, with 80% of HFMD patients having detectable NtAb just one day after disease onset [48]. Maternal NtAb are critical, protecting infants from symptomatic infection, with titers waning by approximately 6 months of age [48]. Despite this, the strength of the humoral response is controversial: NtAb titers in mild HFMD patients do not significantly differ from those in patients with CNS involvement [48,49,50]. B cells, rather than CD4^+^ T cells, were shown to be crucial for protection in mice, suggesting that NAb production can be CD4 T-cell-independent [51]. However, CD4^+^ T cells assist in achieving higher NAb titers [51]. The VP1 antigen is the most immunogenic [52], with VP2-28 identified as a cross-neutralizing epitope [48]. Humoral immunity is associated with the risk of Antibody-Dependent Enhancement (ADE) at sub-neutralizing titers, which is a potential mechanism for high risk in infancy [47]. Age dictates the primary antibody class produced by ASCs (antibody-secreting B cells), with IgG predominating in older children and IgM in younger children [48].

In the ADE mechanism, non-neutralizing antibodies (e.g., IgG) form a complex with the virus, which, instead of neutralization, facilitates viral entry into cells via receptors for the Fc region or complement activation, leading to dissemination and intensified disease. This concept, also referred to as Original Antigenic Sin (OAS) in the context of heterologous infections, must be considered during vaccine design [53,54].

### 4.2. Cellular Response

While neutralizing antibodies are crucial for limiting early viral dissemination, effective viral clearance and control of immunopathology depend on robust T cell responses. The cellular response (CD4^+^ and CD8^+^ T cells) is vital for viral clearance and correlates with prognosis. Cytotoxic CD8^+^ T cells are essential for clearing the infection [49]. After infection, a significant increase in the number of circulating and infiltrating activated T CD4^+^ and CD8^+^ cells (CD69^+^/HLA-DR^+^ markers) is observed [55]. Although B, CD4^+^, and CD8^+^ T cells are detected within the CNS tissue, their role is contradictory: they may help combat infection or contribute to neuropathology. For this reason, the presence of T lymphocytes in the CNS, although sparse compared to macrophages and neutrophils, is strongly associated with an effector function that may lead to neuronal and microglial damage [18].

The T cell profile is often pathogenic: a key issue is the imbalance of Th1/Th2 and Th17/T_reg_ subsets [48,56]. Th17 cells and IL-17A levels are highest in severe HFMD cases [48,55]. Furthermore, elevated Th1 cytokines like IFN-γ, and TNF-α [49] are linked to PE development, where pathologically high IFN-γ contributes to increased vascular permeability. This cytokine dysregulation, characteristic of an uninhibited T cell response, is the main driving force behind cardiopulmonary collapse in severe EV-A71.

### 4.3. Dysfunction of Regulatory T Cells (T_reg_) and cAMP Reduction

The pathological progression is marked by a critical failure of immune regulation. Severe EV71 stages (ANS dysregulation and PE) correlate with a significantly lower frequency of circulating regulatory T cells (T_reg_, specifically CD4^+^CD25^+^Foxp3^+^ cells) compared to mild cases. This depletion impairs the suppression of effector T cells and leads to the uninhibited production of pathological cytokines [57]. The underlying mechanism involves cyclic adenosine monophosphate (cAMP), a key inhibitory second messenger. Patients in the most severe stages (ANS dysregulation/PE) exhibit significantly decreased plasma concentrations of cAMP. This reduction in cAMP activity is associated with a failure to suppress innate immune functions, T cell proliferation, and proinflammatory mediator production [58].

T Follicular Helper cells (T_FH_), marked by PD-1 expression, increase during the acute phase and modulate the humoral response [48,49]. T cells target both structural (VP2) and nonstructural antigens (3D^pol^), with dominant CD4-dependent responses against 3D^pol^ suggesting its protective importance [49]. The susceptibility is further linked to CTLA-4 polymorphisms, a negative T cell regulator [48].

A key mechanism in early defense involves plasmacytoidal dendritic cells (pDCs), which exhibit the highest expression of both SCARB2 and PSGL-1. The virulence of EV-A71 strains is linked to their ability to bind PSGL-1 (VP1-145 A/G/Q strains), which are more frequently associated with severe disease. Importantly, pDCs are activated to secrete protective IFN-α via the PSGL-1 pathway, a process that does not require productive viral replication [59].

## 5. Clinical Implications and Biomarkers of Severe EV-A71 Infection

The clinical severity of EV-A71 infection reflects not only the extent of viral dissemination, but, more importantly, the host’s dysregulated immune response. The following section outlines the key clinical correlates and cytokine biomarkers derived from understanding the host–pathogen interface.

### 5.1. Pathogenic Cytokine Storm and Clinical Correlates

Severe EV-A71-associated neurological disease, particularly brainstem encephalitis (BE) progressing to fatal neurogenic pulmonary edema (PE), is characterized by a rapid, systemic cytokine storm. Cytokines consistently found to be significantly elevated in the serum and cerebrospinal fluid (CSF) of severely ill patients include IL-6, IL-1β, TNF-α, and IFN-γ [60]. The increase in these pro-inflammatory cytokines, particularly pathological high IFN-γ, is linked to PE pathogenesis by increasing pulmonary vascular permeability. Furthermore, IL-6 can leak across an altered blood–brain barrier (BBB) into systemic circulation, driving the development of fatal PE. The highest levels of Th17 cells and their signature cytokine, IL-17A, are strongly associated with severe HFMD cases [61]. This Th17/T_reg_ imbalance serves as a potential biomarker for severe disease progression. Elevated chemokines such as IL-8, IP-10, and MCP-1 in serum and CSF are crucial for predicting severity [60]. The key cytokine and chemokine profiles that distinguish severe EV-A71 infection from mild cases, serving as potential prognostic biomarkers, are summarized in Table 1.

### 5.2. Biomarkers and Predictors of Immune Dysfunction

Analysis of immune cell status and host factors provides valuable insight into disease progression and is essential for developing prognostic tools. Severe cases are characterized by dysfunction and depletion of the monocyte lineage. This impaired immune status is highlighted by a diminished activation status (HLA-DR, CD38^+^) and an increased expression of co-inhibitory markers PD-1/PD-L1 on circulating monocytes, suggesting a state of immune exhaustion [43]. The nuclear protein HMGB1 (High-Mobility Group Box 1), released from activated macrophages and necrotic cells, is a key danger signal. High extracellular levels of HMGB1 correlate with IL-6/TNF-α production and are strong indicators of severe systemic inflammation and lethal endotoxemia [60]. This immunological vulnerability is particularly acute in children under 12 months, a period where developmentally determined innate immune bias leads to the preferential production of high levels of pro-inflammatory cytokines like IL-6 and IL-1β and lower levels of protective Th1-type cytokines. Polymorphisms in host genes regulating immune responses are associated with severity, including those found in the pattern recognition receptor MDA5 and the T-cell negative regulator CTLA-4 [60,61].

## 6. Discussion

### 6.1. Vaccine Efficacy and Real-World Effectiveness

Inactivated EV-A71 vaccines offer significant protection against HFMD, which has been demonstrated by large Phase III clinical trials and post-licensure studies [67]. Estimated efficacy of the two-dose schedule against EV-A71 associated HFMD ranged from 90.0% to 97.9% in clinical trials and remained high at 26 months post-vaccination [67,68,69]. However, real-world effectiveness (VE) is typically lower, estimated at 84.2% for the two-dose schedule due to factors such as heterogeneous populations and deviations from ideal vaccination schedules [67].

The high effectiveness of the vaccine is particularly pronounced against severe outcomes and hospitalizations (up to 100% efficacy), suggesting that the mechanism of protection primarily involves preventing disease progression [67,68,70,71]. This mechanism is hypothesized to involve the reduction in viral load by specific antibodies and memory cells, which lowers the likelihood of EV-A71 crossing the blood–brain barrier [67]. Protection levels have been observed to be higher in older children (aged ≥ 36 months) compared to younger children, correlating with a more mature immune system and higher seroconversion rates [72]. Nonetheless, studies indicate a decline in effectiveness beyond six months post-vaccination, and limited data prevent a formal meta-analysis of long-term protection beyond two years, highlighting a critical gap in public health knowledge [67].

### 6.2. Cross-Protection, Genotype Shift, and Multivalent Strategies

The inactivated EV-A71 vaccines demonstrate strong cross-protection against multiple EV-A71 sub-genotypes (e.g., A, B, C, B4, B5, C2, C4), which enhances their versatility [67,69,71]. However, the vaccines do not confer cross-immunity against other circulating enterovirus serotypes that cause HFMD, such as CVA16, CVA6, and CVA10 [67]. The widespread use of the EV-A71 vaccine has led to significant serotype replacement (virus-type replacement), where the reduction in EV-A71 infection rates has been substantially followed by an increase in non-vaccine strains (CVA6, CVA10), which are now the predominant pathogens in many regions of China [67,68,73]. This shift underscores the need to accelerate the research and development of bivalent and multivalent vaccines (e.g., incorporating CVA6 and CVA10) to maintain the overall preventive effect of the immunization program [67,68,71].

### 6.3. Safety Profile and Immunological Risks

The safety profile of the inactivated EV-A71 vaccines is robust, with only mild, transient adverse events reported in clinical trials and real-world studies [73]. Crucially, the potential risk of Antibody-Dependent Enhancement (ADE) must be considered. ADE, whereby sub-neutralizing concentrations of antibodies enhance viral entry via FcγR receptors expressed on monocytes/macrophages, is a known phenomenon for EV-A71 in in vitro and murine models [69]. However, no evidence of enhanced disease or ADE was observed in large-scale clinical trials of the current vaccines [69]. The use of Intravenous Immunoglobulin (IVIG) may also entail potential risks of ADE, as in vitro studies have shown that insufficient concentrations of IVIG could promote EV-A71 infection [69,74,75]. The continued monitoring of vaccine quality is supported by the development of novel tools, such as the monoclonal antibody NHRI2016–1, which exclusively recognizes effective antigens and correlates in vitro potency with in vivo immunogenicity [71].

### 6.4. Therapeutic Strategies: Risk/Benefit Analysis and Pediatric Safety

Immunomodulation is the primary therapeutic direction for severe EV-A71, as the pathogenesis is substantially driven by uninhibited inflammation rather than viral load alone. However, therapeutic proposals must be weighed against safety concerns specific to the pediatric population. The clinical benefit of Intravenous Immunoglobulin (IVIg) is thought to be mediated primarily by its immunomodulatory properties that suppress the cytokine storm, rather than by neutralizing antibodies alone [76].

The principle of limiting host-mediated damage guides the development of several interventional classes, each posing distinct safety issues, particularly regarding immune reconstitution, the risk of secondary infections, and the limited human evidence for pediatric use.

Cytokine blockade (Anti-IL-6/IL-1Ra), exemplified by interventions targeting the pathogenic IL-6 axis, which reduced mortality in animal models, presents significant risks [77,78]. These interventions are supported predominantly by preclinical evidence, and the systemic suppression of key inflammatory mediators (IL-6, IL-1β) may increase the risk of secondary infections (bacterial/fungal) or compromise essential early viral clearance, potentially prolonging viral replication [79,80,81]. Similarly, the concept of checkpoint modulation (PD-1/PD-L1 blockade), which aims to restore monocyte function, must be treated with caution, as the long-term effects of systemic immune modulation in children are unknown and carry the risk of inducing autoimmunity or exacerbating neuropathology upon rapid immune reconstitution [43,82,83]. Furthermore, antiviral drug development focused on host-factor inhibitors, such as those targeting Prohibitin (PHB), a mitochondrial factor whose inhibition reduces neuropathogenesis, must address severe concerns regarding systemic toxicity. Since PHB is crucial for essential mitochondrial function across all cell types, achieving therapeutic benefit requires highly specific neuronal targeting to avoid widespread organ damage, especially to the cardiovascular system, which is already vulnerable in severe EV-A71 infection [23,84].

Ultimately, any proposed therapeutic intervention, whether involving cytokine blockade, checkpoint modulation, or host-factor inhibition, requires rigorous, context-specific risk/benefit analysis. This assessment is particularly crucial for pediatric viral infections, where current preclinical evidence supporting these novel approaches often fails to address the potential harms associated with immune reconstitution, the risk of secondary infections, and timing/dosing challenges unique to the developing human immune system.

## 7. Conclusions

The host immune response to Enterovirus A71 (EV-A71) demonstrates that disease severity arises not merely from viral replication, but from dysregulated host immunity—particularly in immunologically immature children. The initial innate response, though rapid, is both intense and maladaptive. Viral proteases effectively subvert key antiviral pathways, while developmental immune bias in infants amplifies proinflammatory signaling and predisposes to immunopathology. Progression to life-threatening complications, such as neurogenic pulmonary edema (PE), is driven by a systemic cytokine surge and failure of adaptive regulation. Although neutralizing antibodies appear quickly, their titers do not correlate with clinical outcome. Instead, prognosis depends on cellular immunity, notably Th17/Treg imbalance and immune exhaustion marked by PD-1/PD-L1 expression on monocytes.

These insights underscore that future therapeutic and prophylactic strategies must extend beyond antibody-mediated protection. Emphasis should be placed on targeted immunomodulation (particularly of the IL-6 and IL-1β axis) and on vaccines that elicit balanced cellular immunity capable of preventing destructive inflammation and reducing long-term neurological sequelae. Understanding these immune mechanisms offers a foundation for more effective, precision-based approaches to EV-A71 control.

## Figures and Tables

**Table 1:** Prognostic Immune Biomarkers and Immunopathological Mechanisms Correlated with Severe Enterovirus A71 (EV-A71) Infection.

Biomarker	Specific Marker	Correlation with Severe HFMD/CNS Involvement	Sample Type	Study Type	Pathological Implication	Supporting References
Th17/Th Cytokines	IL-17A	Highest levels observed in severe cases.	Serum, Plasma, Peripheral blood mononuclear cells (PBMCs)	Cohort study, case–control study, case–control study	Indicator of critical Th17/Treg imbalance and severe inflammatory promotion.	[62,63,64]
	IFN-γ	Elevated levels linked to PE pathogenesis.	CSF	Case–control study	Contributes to increased pulmonary vascular permeability.	[38]
Proinflammatory Cytokines	IL-6, IL-1β, TNF-α	Consistently and significantly elevated in CSF/serum in severe/fatal cases.	Serum, serum	Case-control study, cross-sectional clinical case–control study	Primary mediators of systemic cytokine storm and neuroinflammation.	[65,66]
Chemokines/Recruitment	IL-8, IP-10, MCP-1	Crucially elevated in serum and CSF.	Serum	Cross-sectional clinical case–control study	Promotes neutrophil and monocyte recruitment to sites of infection and neurological injury.	[65]
Regulatory Signaling	cAMP	Significantly decreased plasma concentration in severe cases.	Plasma, CSF	Case–control study	Indicator of failed inhibitory signaling (PDE pathway dysregulation) leading to uninhibited T cell activation.	[58]
Immune Dysfunction	Treg (CD4+CD25+Foxp3+)	Significantly lower frequency observed in severe stages.	Peripheral blood mononuclear cells (PBMCs)	Case–control study	Critical loss of immune control (suppression) over the inflammatory response.	[58]
	Monocytes (PD-1/PD-L1)	Increased expression of co-inhibitory markers.	Peripheral blood mononuclear cells (PBMCs)	Case–control study	Sign of immune exhaustion and reduced functional capacity.	[43]

## Data Availability

No new data were created or analyzed in this study.
